# Susceptibility to Pitting Corrosion of AerMet 100 and 4340 Alloys for Aeronautical Applications

**DOI:** 10.3390/ma19071397

**Published:** 2026-03-31

**Authors:** Miguel Sergio Huerta-Zavala, Citlalli Gaona-Tiburcio, Demetrio Nieves-Mendoza, Jesus Manuel Jaquez-Muñoz, Jose Cabral-Miramontes, Erick Maldonado-Bandala, Rene Croche-Belin, Miguel Angel Baltazar-Zamora, Laura Landa-Ruiz, Luis Daimir Lopez-Leon, Javier Olguin-Coca, Facundo Almeraya-Calderon

**Affiliations:** 1 Universidad Autónoma de Nuevo León, FIME, Centro de Investigación e Innovación en Ingeniería Aeronáutica (CIIIA), San Nicolás de los Garza 66455, Mexico; miguel.huertaz@uanl.edu.mx (M.S.H.-Z.); citlalli.gaonatbr@uanl.edu.mx (C.G.-T.); jose.cabralmr@uanl.edu.mx (J.C.-M.); facundo.almerayacld@uanl.edu.mx (F.A.-C.); 2 Facultad de Ingeniería Civil/Facultad de Ingenieria Mecánica y Eléctrica, Universidad Veracruzana, Xalapa 91000, Mexico; dnieves@uv.mx (D.N.-M.); rcroche@uv.mx (R.C.-B.); mbaltazar@uv.mx (M.A.B.-Z.); lalanda@uv.mx (L.L.-R.); 3 Centro de Ciencias de la Ingeniera, Universidad Autonóma de Aguascalientes, Aguascalientes 20340, Mexico; jesus.jaquez@edu.uaa.mx; 4 Área Académica de Ingeniería y Arquitectura, Universidad Autónoma del Estado de Hidalgo, Carretera Pachuca-Tulancingo Km. 4.5, Pachuca de Soto 42082, Mexico; luis_lopez@uaeh.edu.mx

**Keywords:** localized corrosion, electrochemical noise, pitting

## Abstract

In the aeronautics industry, high-strength steels such as AerMet 100 and 4340 are widely used in critical structural components that must withstand extreme operating environments. These materials possess high tensile strength, fracture toughness, and fatigue resistance. The aim of this investigation is to study the susceptibility to localized pitting corrosion of two aeronautics alloys, AerMet 100 and 4340, and their immersion in H_2_SO_4_, NaCl, and HCl solutions at room temperature, using electrochemical noise (EN) according to the ASTM ASTM-G199 standard. The EN signal was filtered by two different methods, and the polynomial method was employed to obtain Rn, LI, Kurtosis, Skewness, and the potential spectral density analysis (PSD). Results indicate that AerMet 100 exhibits lower corrosion rate—up to an order of magnitude lower than 4340. The resistance noise of 1599 Ω·cm^2^ in NaCl is higher. This same behavior is replicated when analyzing the noise impedance response (Z_n_). In conjunction with the analyses of PSD slope, it is reported that localized corrosion is the predominant mechanism in the evaluated environments.

## 1. Introduction

Corrosion in the aerospace industry, as in many other industries, is a significant safety concern. Therefore, mechanical strength, as well as corrosion and oxidation resistance, must be optimized for each specific application in this industry [1,2]. According to Findlay et al. [3], pitting corrosion can generate localized stress concentrations, acting as a trigger for component failure.

For aeronautical components, such as landing gear, arresting hooks, catapult launch bars, and others, steels like AerMet 100 and 4340 have been used. These steels have good mechanical properties, but their behavior in various corrosive environments requires further study using electrochemical techniques for evaluation.

The literature review revealed a scarcity of reported studies on electrochemical noise techniques for AerMet 100 and 4340 alloys [4,5,6,7]. The available works that use electrochemical tests focus exclusively on their behavior under stress-assisted corrosion (SCC), cyclic potentiodynamic polarization curves (CPP), and electrochemical impedance spectroscopy (EIS). Lee et al. [8] measured corrosion rates using immersion and salt spray tests and concluded that AerMet 100 exhibits a significant advantage in corrosion resistance compared to 300M steel. Similarly, a year later, using the sodium chloride open-circuit potential technique, they determined that AerMet 100 exhibits a more noble potential than 300M steel [9].

Using salt spray, polarization, and EIS techniques, Sun et al. [10] concluded that the corrosion resistance and corrosion protection capabilities of AerMet 100 steel are superior to those of 300M steel. Zhong et al. [11] subjected AerMet 100 to CPP after exposure to a salt spray test and concluded that the passive region of AerMet 100 steel is more extensive and exhibits high resistance to passivity breakdown and pitting. However, they note that AerMet 100 steel is highly susceptible to chloride-ion-induced passivity breakdown. On the other hand, Diaz et al. [12] used the polarization curve technique to determine the E_corr_ and i_corr_ in AISI 4340 with a duplex pulsed plasma nitriding process at different times. Using EIS, Yıldız and Gerengi [13] indicate that 4340 exhibits greater resistance than 4140 and 5140 in sodium chloride, and [14] they used CPP to compare the corrosion resistance of 4340 in sodium chloride with two quenching methods. PPC experiments show that increasing surface roughness increases steel corrosion by increasing the alloy’s corrosion current. Electrochemical impedance spectroscopy (EIS) diagrams also indicated that an increase in R_ct_ reduces the alloy’s corrosion resistance. Pitting corrosion was observed using scanning electron microscopy (SEM); these tests were performed in a 3.5 wt. % NaCl environment. In other studies, AerMet 100 and 4340 steels have been coated and evaluated using electrochemical and accelerated corrosion techniques, such as CPP, EIS, and salt chamber testing [15,16,17].

Electrochemical tests are fundamental tools for studying material corrosion, as they enable rapid, precise evaluation of the material’s interaction with its environment. Electrochemical noise (EN) has become a fundamental tool in corrosion science, primarily for detecting initial stages of localized corrosion and for being a test that does not alter the material’s condition. Its principle is based on spontaneous fluctuations in potential and current at the material–electrolyte interface. This technique allows the determination of corrosion rate, resistance, and mechanism [18,19,20,21,22,23].

Electrochemical noise analysis can be performed using three methods: time domain, frequency domain, and time–frequency domain. Time domain analysis visually examines current and potential signals, known as transients, and enables the identification of characteristic corrosion behaviors. Within this domain is statistical analysis, which, using parameters such as current density (i_corr_), noise resistance (R_n_), and localization index (LI), supplemented with Skewness and Kurtosis, can identify the type of corrosion and its severity [24,25].

On the other hand, frequency domain analysis requires transforming time domain signals into the frequency domain. The most widely used method is the fast Fourier transform (FFT), which yields the potential spectral density (PSD), current, and power [26,27]. One way to analyze PSD is through spectral slope, which, according to criteria established by Legat and Doleček [26], can be associated with specific corrosion mechanisms. This analysis allows us to determine the impedance (Z_n_), which, like resistance, helps us establish the severity of the attack. Several authors have used these techniques to study the corrosive behavior of different metallic materials [28,29,30,31,32].

This work aims to study the electrochemical behavior of localized pitting corrosion of AerMet 100 and 4340 aeronautical steels using electrochemical noise analysis in H_2_SO_4_, NaCl and HCl solutions at room temperature. The microstructural analysis and corrosion morphology was obtained by scanning electron microscopy (SEM). This type of investigation seeks to replicate localized pitting corrosion behavior of exposed steels in the chemical and chloride conditions found in marine and industrial environments, in aeronautic components such as landing gear, arresting hooks, catapult launch bars, and others.

## 2. Materials and Methods

### 2.1. Materials

The AerMet 100 and 4340 steels used were commercial steels in the form of cylindrical bars 1 in diameter. They were employed and tested in the (quenching and tempering) as-received condition.

The AerMet 100 and 4340 steels used in this research belong to the high- and ultra-high-strength steel category, used for aeronautical, structural, and defense applications where high mechanical properties, high toughness, and metallurgical reliability are required. AerMet 100 is an ultra-high-strength martensitic steel with a minimum tensile strength of 1930 MPa and a fracture toughness greater than 100 ksi. It is primarily used in landing gear, jet engine shafts, and structural components [33]. AISI 4340, on the other hand, is a nickel-chromium-molybdenum steel recognized for its toughness, strength, and hardenability, with a tensile strength of 930–1080 MPa. It is also used in the manufacture of gears, power-transmission shafts, landing gear, and high-stress structural components [34].

The chemical composition of both steels is presented in Table 1 and was determined by X-ray fluorescence.

### 2.2. Microstructural Characterization

The alloys were prepared by metallographic techniques following ASTM E3-95 [35], using a series of silicon carbide abrasives with grit sizes ranging from 120 to 4000, polished with 0.1-micron alumina. The microstructure was revealed using a 5 wt. % Nital solution. Finally, the microstructural analysis was carried out by scanning electron microscopy (SEM, JEOL-JSM-5610LV, Tokyo, Japan) at 2000× magnification. A secondary electron (SE) detector was used to investigate the microstructure of the samples. The samples tested for corrosion were observed by SEM.

### 2.3. Electrochemical Technique

**Figure 1:**
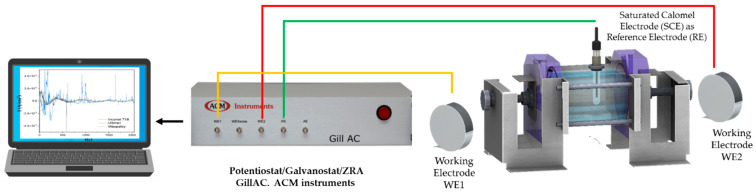
Three-electrode cell for electrochemical noise (EN) measurements [37].

The corrosion behavior of the AerMet 100 and 4340 alloys was determined using electrochemical noise analysis. The samples were exposed to three electrolytes: 1 wt. % H_2_SO_4_, 1 wt. % HCl, and 3.5 wt. % NaCl solutions, at room temperature, with an exposed working area of 1 cm^2^. The tests were performed using three-electrode cell testing [AERMET 100 and 4340 steels served as working electrodes (WE1), a saturated calomel reference electrode (RE) was used; a platinum mesh was used as the counter electrode (WE2)]; see Figure 1. And the electrolytes were not deaerated; a Potentiostat/Galvanostat/ZRA (Zero Resistance Ammeter) (Solartron 1287A, Bognor Regis, UK) was used. The samples were polished with a SiC grit paper until grade 600, followed by ultrasonic cleaning in ethanol and deionized water for about 10 min each.

Electrochemical measurements were performed as follows: The electrochemical noise technique was performed according to ASTM G199 [36], with 1024 data points per test at an acquisition rate of 1 data point per second. The current and potential electrochemical noise was monitored with respect to time for each electrode–electrolyte combination under open-circuit conditions. The current and potential time series were visually analyzed to interpret the signal transients and define the behavior of the frequency and amplitude of fluctuations as a function of time. Statistical data were processed using a MATLAB 2025a program developed by the Corrosion and Protection Group of CIIIA/FIME/UANL, which has been employed in past research [31]. All results were obtained in duplicate. Time domain data allows the calculation of statistical measures such as R_n_, LI, Kurtosis, and Skewness. The electrochemical noise (EN) signal, after the DC (DC) component was removed using a ninth-degree polynomial, was analyzed; this polynomial degree was selected due to the fact that at the 5th and 7th filter, the EN signal still presents DC, so to eliminate the signal, a 9th polynomial filter was the best option for those materials. The PSD (power spectral density) data were processed with a Hann window before being transformed to the frequency domain using FFT (fast Fourier transform).

## 3. Results and Discussion

### 3.1. Microstructure by SEM

**Figure 2:**
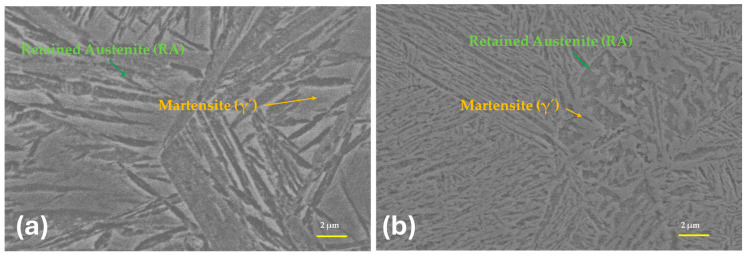
SEM-SE, microstructures of (**a**) 4340, (**b**) AerMet 100 4000×.

The microstructures of the initial samples were analyzed by SEM. Figure 2 shows the microstructure of 4340 and AerMet 100 steels. Figure 2a shows a plate tempered martensite microstructure and traces retained austenite [38,39], which provides high hardness and mechanical strength. Figure 2b shows the microstructure of AerMet 100 steel, which is a fine martensite microstructure with retained austenite, which was obtained from thermal control during quenching. This microstructure contributes to high toughness and fracture resistance [40,41].

### 3.2. Electrochemical Noise

#### 3.2.1. Time Domain Analysis

Electrochemical noise (EN) is used to assess corrosion activity by simultaneously recording spontaneous potential and current fluctuations. The EN signal consists of three components: a random component, a steady-state component, and a direct current component. This technique can be interpreted differently by various authors [42,43,44,45,46].

To calculate noise resistance (Rn), the standard deviation from time series data must be determined. These statistical parameters offer insights into corrosion mechanisms and kinetics. Research by Turgoose and Cottis [47] reveals that higher corrosion rates correspond to increases in variance and standard deviation. Use Equation (1) to compute the standard deviation (σ), standard deviation of the potential data (σ_v_), standard deviation of the current data (σ_I_), working electrode area (A) and derive Rn (Equation (2)), utilizing EN time series information (EPN and ECN) as the foundation.

(1) $$\sigma_{x}=\sqrt{\bar{x^{2}}}=\sqrt{\frac{\sum_{1}^{N}{\left(x_{i}-\bar{x}\right)}^{2}}{N}}$$

(2) $$R_{n}=\frac{\sigma_{v}}{\sigma_{I}}\times A$$

Since R_n_ and R_p_ are correlated, the Stern–Geary equation (Equation (3)) can be applied as an analog relation to evaluate corrosion kinetics [48]. B is a constant with a recommended value of 0.026 V for active and 0.052 V for passive corrosion.

(3) $$i_{corr}=\frac{B}{R_{n}}$$

The localization index (LI), defined by Equation (4), is a parameter used to estimate, as a first approximation, the type of corrosion occuring in a given system [49,50,51,52]. LI values approaching zero indicates uniform (general) corrosion; values in the range fron 0.01 to 0.1 indicate mixed corrosion, whereas values from 0.1 to 1 correspond to pitting corrosion.

(4) $$LI=\frac{\sigma_{I}}{I_{RMS}}$$

Where σ_I_ is the standard deviation of the current data and *I_RMS_* is the root mean square value of the corrosion current noise.

In this study, the analysis of Kurtosis and Skewness was included to determine the type of corrosion, as the localization index had limitations, as noted in 1995 by Mansfeld and Sun [51], and should therefore be used with caution. However, in the patent of Reid and Eden [52], they indicate that the third and fourth statistical moments, Skewness and Kurtosis (see Equations (5) and (6)), can be used to determine the type of corrosion [53,54,55,56,57], where N is the number of data studied and x is the EN signal.

(5) $$Skewness=\frac{1}{N}\sum_{i=1}^{N}\frac{{{(x}_{i}-\bar{x})}^{3}}{\sigma^{3}}$$

(6) $$Kurtosis=\frac{1}{N}\sum_{i=1}^{N}\frac{{{(x}_{i}-\bar{x})}^{4}}{\sigma^{4}}$$

The following table shows the relationship between the Kurtosis and Skewness values used to determine the material’s corrosion type (Table 2).

**Figure 3:**
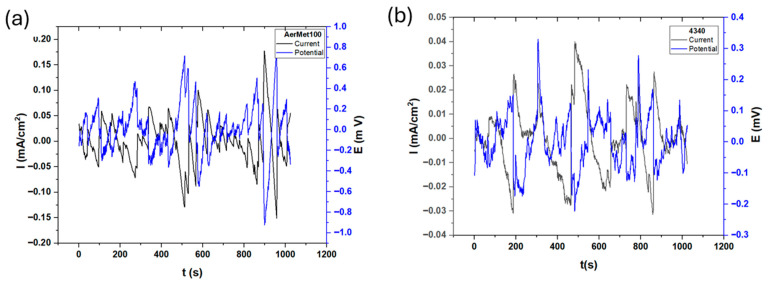
Electrochemical current and potential noise time series for AerMet 100 (**a**) and 4340 (**b**), exposure to H_2_SO_4_ solution.

Figure 3 presents the electrochemical potential and current noise of the steels exposed to sulfuric acid. In the electrochemical noise analysis for potential, AerMet 100 showed a slightly higher amplitude of 0.8 mV compared to 4340, which recorded 0.35 mV. This suggests that 4340 exhibits a more stable behavior with lower variation in charge transfer between the metal and the electrolyte. The R_n_ values are 5.44 and 6.015 Ω·cm^2^ for AerMet 100 and 4340, respectively (see Table 3). The LI is associated with uniform corrosion, indicating that corrosion occurs uniformly at the surface. The results obtained by Skewness are the same as those for uniform corrosion. The Electrochemical Current Noise (ECN) signal of AerMet from Figure 3a shows how high-frequency transients increase in amplitude when time increases. This means the beginning of pitting nucleation, between seconds 0–200, the current increases after second 600, indicating an increase in the corrosion kinetic and the localization of the corrosion process. Figure 3b in ECN shows a behavior related to localized corrosion with the steps in seconds 200, 300, 500, 700, 900 and 1000. That behavior is related to the generation of a pseudo-passive layer by the decrease in the current, but the anodic transients that occur in those seconds are due to pseudo-passive layer breaking, regeneration and breaking.

**Figure 4:**
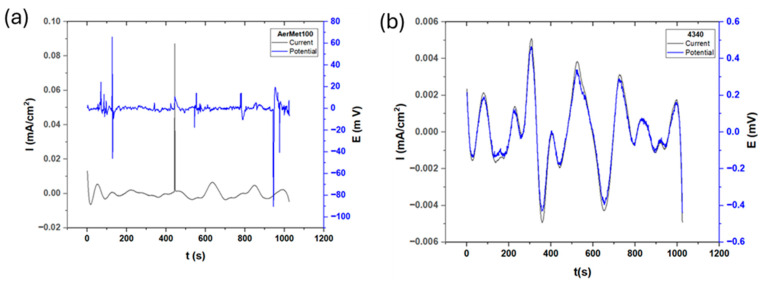
Electrochemical current and potential noise time series for AerMet 100 (**a**) and 4340 (**b**) exposed to NaCl solution.

Figure 4 shows the electrochemical noise series of potential and current for both steels exposed to a sodium chloride solution. Figure 4a illustrates the electrochemical response of AerMet 100, where minimal fluctuation activity and transient events in current are observed, with only one significant transient occurring at approximately 430 s. This transient exhibited high amplitude but short duration, likely associated with the nucleation of a localized pit; however, the material was able to repassivate rapidly. A similar trend is observed in potential noise analysis.

A notable difference between the steels exposed to sodium chloride and those tested in previous environments is the resulting corrosion current density.

**Figure 5:**
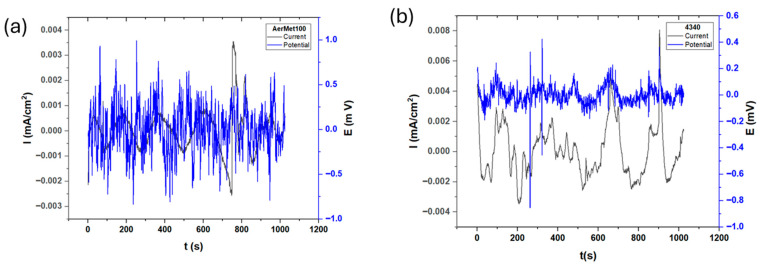
Electrochemical current and potential noise time series for AerMet 100 (**a**) and 4340 (**b**), exposure to HCl solution.

Figure 5 shows the electrochemical noise results for the steels exposed to hydrochloric acid. Unlike the behavior observed in other electrolytes, both alloys exhibit chaotic responses characterized by short-lived anodic and cathodic transients in potential and current noise, indicating repeated breakdowns of the passive film in localized areas and, consequently, increased material dissolution. The current shows sinusoidal behavior; the Rn of AerMet 100 is 1599.84 Ω·cm^2^ and it is 87.79 Ω·cm^2^ for 4340, indicating it is more corrosion-resistant than AerMet. It occurs due to the possible formation of a possible passive film at the surface, which increases noise resistance. The corrosion type of materials is predominantly uniform, which can be related to the microstructure. The Electrochemical Current Noise signal from Figure 4a shows behavior of AerMet 100. In current noise, it shows a periodic behavior with little high-frequency, low-amplitude transients. The potential noise signal shows a great number of anodic and cathodic transients, indicating that the material present has localized corrosion. Figure 4b shows the behavior of 4340, where the current noise presented anodic transients, indicating localized corrosion. Those transients occurred in seconds 100, 150, 270, 690, 900 and 1100.

#### 3.2.2. Frequency Domain Analysis

For PSD analysis, it is necessary to transform the time domain EN to the frequency domain using FFT, since the EN signal (with a polynomial filter applied) is correlated, and then calculate the spectral density using Equations (7) and (8) [58,59].

(7) $$R_{xx}\left(m\right)=\frac{1}{N}\sum_{n=0}^{N-m-1}x\left(n\right)\cdot x\left(n+m\right),when\;values\;are\;from\;0<m<N$$

(8) $$\Psi_{x}\left(k\right)=\frac{\gamma \cdot t_{m}}{N}\cdot \sum_{n=1}^{N}\left(x_{n}-{\bar{x}}_{n}\right)\cdot e^{\frac{{-2\pi kn}^{2}}{N}}$$

where R_xx_ (m) is the autocorrelation of x(n) process (noise signal) for a delay (lag) m. N means the sample number. In Equation (7), ψ_x_(k) means the PSD of x signal in binary frequency k, γ is the correlation factor or normalization, usually between 1 and 2. Tm is the sampling intervale. E^−j2πkn2/N^ is the relation of a complex exponential of discrete Fourier transform (DFT) that changes a time signal into a frequency signal.

The interpretation of PSD is based on the limit frequency-to-cut frequency ratio, with the cut frequency indicating when a slope began and ended, because a slope can help identify the corrosion mechanism. Cut frequency provides information about the sample’s representation after pitting [60]. Slope is defined by βx, and is represented in Equation (9):

(9) $$log\Psi_{x}=-\beta_{x}\log f$$

The frequency zero limit (ψ^0^) provides information on material dissolution because the power PSD is related to the total amount of energy present in the system [61]. It is important to clarify that material dissolution is only present in the current PSD [62,63,64]. The next table, proposed by Mansfeld et al. [51] in 1998 to determine the corrosion phenomena occurring on the material surface, is adapted to decibels, see Table 4 [65].

It is important to emphasize that some values are the same for the two types of corrosion; this could provide another way to study the slope as a function of frequency [64].

**Figure 6:**
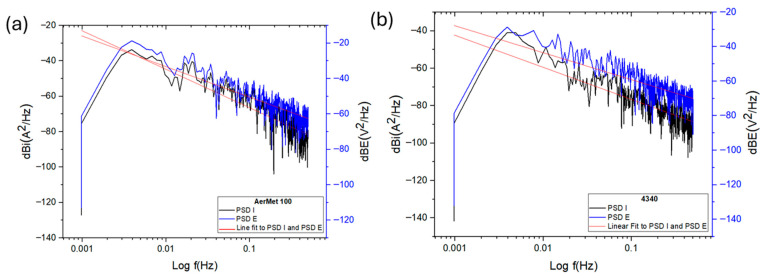
Power spectral density (PSD) in potential and current for AerMet 100 (**a**) and 4340 (**b**), exposure to H_2_SO_4_ solution.

In power spectral density (PSD) plots, the vertical position of the curves is related to the magnitude of corrosive activity; higher values indicate greater current density demand, which corresponds to increased electrochemical activity. Figure 6 shows the PSD slopes for current and potential of AerMet 100 and 4340 alloys—Figure 6a and Figure 6b, respectively. Both alloys exhibit similar behavior across the frequency range, with comparable current demand and slope values: −16.29 dB·(A^2^/Hz) for AerMet 100 and −12.85 dB·(A^2^/Hz) for 4340 (see Table 5).

**Figure 7:**
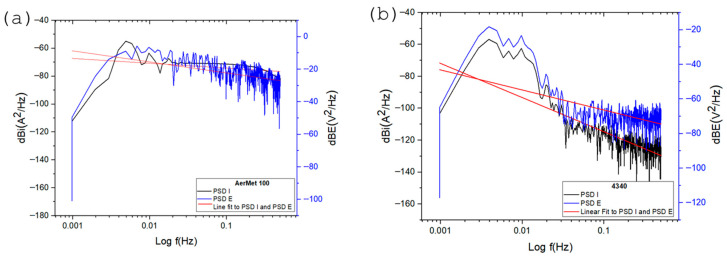
Power spectral density (PSD) in potential and current for AerMet 100 (**a**) and 4340 (**b**), exposure to NaCl solution.

Figure 7 shows the PSD slopes of the alloys exposed to sodium chloride, which exhibit different behavior compared to each other and to those exposed to sulfuric acid. Notably, a decrease in current demand was observed, with more negative dB values compared to the sulfuric acid environment. This indicates that the alloys exposed to sodium chloride exhibit lower corrosion kinetics.

**Figure 8:**
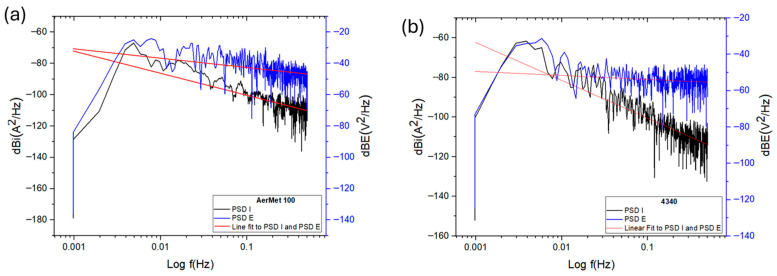
Power spectral density (PSD) in potential and current for AerMet 100 (**a**) and 4340 (**b**), exposure to HCl solution.

Figure 8 illustrates the electrochemical behavior of the steels exposed to hydrochloric acid, which is similar to that observed in sodium chloride in terms of current density demand. Both alloys exhibit comparable values and PSD slopes close to each other. AerMet 100 shows a slope of −10.54 dB·(A^2^/Hz), indicating pitting behavior. In comparison, the 4340 alloy presents a slope of −14.30 dB·(A^2^/Hz), also associated with localized corrosion.

The noise impedance, Z_n_ (f), also called spectral noise resistance, is defined (Equation (10)) as [66,67]:

(10) $$Z_{n}=\sqrt{\frac{\psi_{V}(f)}{\psi_{I}(f)}}$$

Noise impedance is calculated by the square root of the PSD divided by the potential PSD and current. The electrochemical noise impedance is related to corrosion resistance, and the inverse is related to conductance and corrosion rate [68,69,70,71].

**Figure 9:**
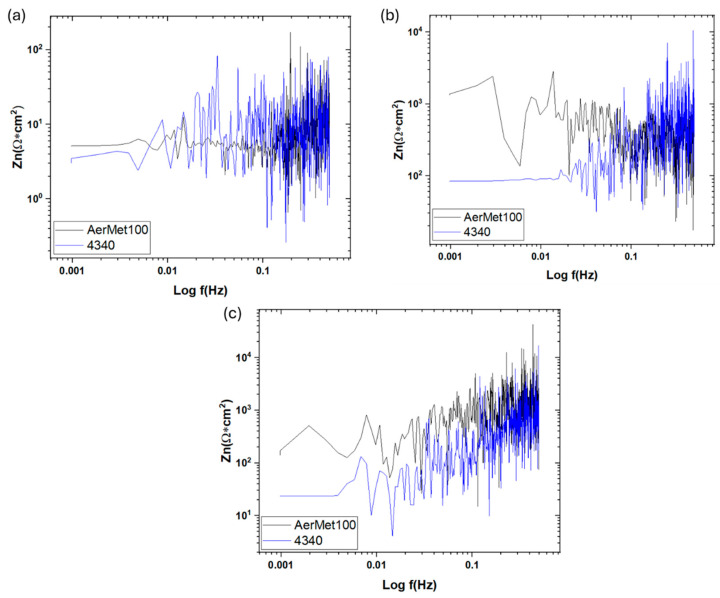
Spectral noise resistance (Z_n_) for AerMet 100 and 4340 steels exposed to H_2_SO_4_ (**a**), NaCl (**b**), and HCl (**c**) solutions.

Figure 9 shows the Z_n_ results for H_2_SO_4_ (a), NaCl (b), and HCl (c). The steels exposed to sulfuric acid exhibit the lowest impedance values among other solutions, with AerMet 100 showing a noise impedance of 10.2 Ω·cm^2^ and 4340 showing 11.46 Ω·cm^2^, as indicated in Table 5. In NaCl, the 4340 presented a value of 570.15 Ω·cm^2^, and AerMet 100 presented 436.74 Ω·cm^2^. In HCl, AerMet 1000 showed a higher noise impedance of 1574.74 Ω·cm^2^, whereas 4340 showed 715.4 Ω·cm^2^.

Table 5 summarizes the electrochemical parameters obtained from both PSD and impedance analyses. Based on current slope values, most steels exhibit pitting corrosion. This information complements the time domain statistical analysis, which indicates that most samples exhibit uniform corrosion.

The Z_n_ response shows a trend analogous to that of the R_n_ values obtained from time domain analysis. Both parameters behave similarly in sulfuric acid and hydrochloric acid solutions. In contrast, in a NaCl solution, the trend is reversed, with AerMet 100 exhibiting lower resistance in the corrosive system. Overall, it can be inferred that the alloys experience the most aggressive attack in sulfuric acid, followed by sodium chloride, and finally hydrochloric acid.

### 3.3. SEM After Electrochemical Noise Measurements

**Figure 10:**
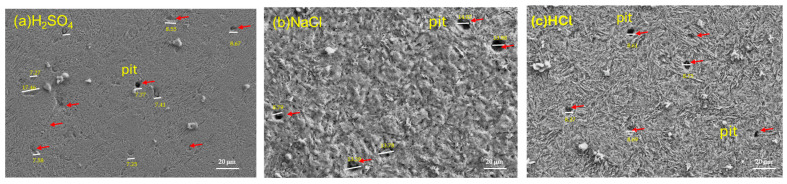
SEM-SE morphology for AerMet 100 steel exposed to H_2_SO_4_ (**a**), NaCl (**b**), and HCl (**c**) solutions.

**Figure 11:**
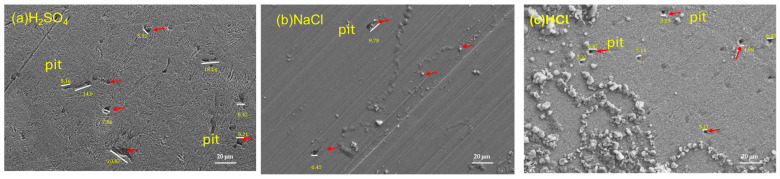
SEM-SE stereoscope morphology for 4340 steel exposed to H_2_SO_4_ (**a**), NaCl (**b**), and HCl (**c**) solutions.

Following electrochemical corrosion measurements, the surface morphology of each sample was examined using scanning electron microscopy. SEM analysis revealed localized pitting corrosion on all steel surfaces (Figure 10 and Figure 11). The steels under study have a pitting density of type A according to ASTM G46 [72]. AerMet 100 steel has an average pit size of: H_2_SO_4_ (7.83 μm), NaCl (12.77 μm) and HCl (8.60 μm). On the other hand, the average pit size of 4340 steel is: H_2_SO_4_ (12.10 μm), NaCl (8.11 μm) and HCl (7.01 μm). The largest pitting size is observed in AerMet 100 steel in the presence of NaCl, followed by 4340 steel in the presence of H_2_SO_4_.

## 4. Discussion

The ECN signal disturbances, which are low-level fluctuations in the corrosion potential between two nominally identical electrodes, is closely linked to electrochemical kinetics [73]. Figure 3, Figure 4 and Figure 5 illustrate this behavior: alloys exposed to a H_2_SO_4_ solution (Figure 3) exhibit a mirror-type response, with current transients occurring simultaneously with potential transients, indicating the formation and breakdown of the oxide layer [32,74,75]. With exposure to NaCl and HCl (Figure 4 and Figure 5) solutions, a more chaotic pattern emerges, with numerous short-lived transients of low amplitude, suggesting pit nucleation, growth, and repassivation, as described by Cottis [22]. AerMet 100 exhibits Type 1 transients [74,75], implying slower repassivation in some cases. This behavior is more pronounced in the presence of Cl^−^ ions.

Localization index analysis suggests uniform corrosion for all samples; however, this method may have limitations, as Masfeld mentioned [51]. Statistical analysis indicates that, in most cases, the corrosion type does not match. This inconsistency is attributed to LI variability. Researchers such as Eden and Mansfeld [51,52,58] have noted that statistical approaches have certain limitations, particularly because Eden introduced the LI years earlier as a means of identifying corrosion types. Therefore, the use of the LI to classify corrosion should be applied with judgment. Additionally, the signal analyzed using the statistical method should not contain a DC component, as removing it reduces the standard deviation and yields more accurate results. In some cases, the results obtained by LI do not correspond to Skewness and Kurtosis; therefore, it is necessary to use other methods of analysis, such as frequency domain and frequency–time.

Therefore, Skewness and Kurtosis were used to refine the interpretation, revealing that while most samples exhibit uniform corrosion, some show localized pitting. The samples that presented two different corrosion types can be associated with the predominance of one of them.

The statistical approach indicates that, in most cases, the corrosion type could not be reliably identified. This limitation is mainly attributed to the variability of the localization index (LI). Several authors, including Eden and Mansfeld, have reported that statistical analyses present inherent limitations, particularly because the LI was originally proposed by Eden years earlier solely as a parameter for corrosion characterization rather than definitive corrosion-type identification. Consequently, LI should be applied with caution when used to infer corrosion mechanisms. In addition, for statistical analysis to yield more accurate and specific results, the analyzed signal must be free of a DC component, as its presence increases the standard deviation and reduces result reliability [76,77].

Other statistical parameters, such as Kurtosis and Skewness, have demonstrated greater reliability. In certain cases, such as with 304 stainless steels, these methods produced results consistent with those obtained using HHT and FFT analyses. However, neither Kurtosis nor Skewness provides a definitive value for passive systems. Among the two, Skewness offers greater accuracy than Kurtosis. Nevertheless, several authors, including Cottis, Turgoose, Jáquez, and Sánchez-Amaya [31,46,52], recommend using Skewness cautiously due to the same inherent limitations associated with statistical methods.

The results of the frequency domain analysis allowed us to obtain the slope parameter values used to identify the corrosion type, which differ from those obtained through the statistical method. This discrepancy is a crucial factor to consider when interpreting slope analyses, though it comes with certain limitations. Nonetheless, examining how the slope changes across different PSD frequencies is essential, as these variations reflect shifts occurring within the corrosion process [75].

PSD slope magnitude can be considered a parameter for distinguishing corrosion type [29,32,65]. All slopes formed from 0.01 Hz, and statistical analysis indicates pitting corrosion for most alloys, except AerMet 100 in NaCl, which showed general corrosion. Similarly, steels in NaCl and HCl exhibited lower current densities than sulfuric acid, which showed the highest values. This is reinforced by noise impedance: H_2_SO_4_ produced the lowest Z_n_ and highest current density, with AerMet 100 consistently outperforming 4340 in all electrolytes.

AerMet 100′s superior resistance, as shown in EN, is attributed to its chemical composition. Elements such as Cr, Ni, and Mo enhance corrosion resistance by forming protective oxides [75,76]. For instance, molybdenum improves pitting resistance by forming a passive MoO_2_ film that acts as an effective barrier against diffusion and reduces active dissolution current [77]. Therefore, upon contact with the electrolyte, AerMet tends to form an oxide layer, which reduces the corrosion rate. However, localized corrosion is still evident, as indicated by the EN results.

Lee et al. [78] compared SCC behavior of AerMet 100, 4340, and 300M, finding AerMet 100 exhibits greater resistance to fatigue and cracking in 3.5 wt. % NaCl, reinforcing earlier results by Lee et al. [79]. Tian et al. [80], using SEM, observed that SCC crack initiation sites correspond to corrosion pits, supporting the conclusion that AerMet 100 develops fewer pits than 4340 under SCC conditions, making it more resistant to this corrosion mechanism, as reflected in the tables presented in this study, where the AerMet 100 presented a higher resistance to corrosion when EN was the characterization technique.

Electrochemical characterization of the AerMet 100 and 4340 steels could have potential applications in the aeronautical industry, such as aircraft landing gear. The structural components of aircrafts made with these steels are exposed to different atmospheres: industrial [acid rain (H_2_SO_4_)] and marine (NaCl). AerMet 100 and 4340 steels may be susceptible to low-temperature localized corrosion when aircrafts are on the floor.

## 5. Conclusions

After studying the electrochemical behavior of localized pitting corrosion in AerMet 100 and 4340 aeronautic steels using electrochemical noise analysis in different electrolytes at room temperature, the research results indicate that:

- EN analysis using asymmetry and the LI show that the system is dominated by a uniform corrosion discrepancy that can be associated with perturbation of the electrochemical noise signal transients.
- EN analysis confirms that AerMet 100 exhibits higher noise resistance and lower corrosion rate—up to an order of magnitude lower than 4340. The resistance of 1599 Ω·cm^2^ in NaCl is higher.
- Frequency domain analysis supports the findings from EN in the time domain. Z_n_ results indicate that AerMet 100 has greater corrosion resistance.
- Alloys exposed to H_2_SO_4_ exhibit the highest corrosion rates and the most aggressive attack as measured by electrochemical noise. Combined with Rn and Zn results, AerMet 100 consistently demonstrates superior noise resistance and a lower corrosion rate than 4340, attributed to its higher Cr, Ni, and Mo content.
- Frequency domain analysis indicate that both AerMet 100 and 4340 steels are susceptible to localized corrosion in the studied solutions, albeit to varying degrees.
- The results indicate that, after corrosion measurements, the surface morphology observed on the steels under study using scanning electron microscopy (SEM) confirm the presence of pitting, representing localized corrosion.

## Figures and Tables

**Table 1:** Chemical composition of different steels (wt. %).

Steels	Cr	Cu	Fe	Mn	Ni	Si	Co	Mo
4340	0.87	0.169	95.88	0.86	1.73	0.2	–	0.251
AerMet	3.16	0.19	71.36	0.06	8.32	–	15.53	1.364

**Table 2:** Corrosion types are evaluated by Kurtosis and Skewness [28].

Corrosion Type	Potential		Current	
	Skewness	Kurtosis	Skewness	Kurtosis
Uniform	<±1	<3	<±1	<3
Pitting	<−2	>>3	>±2	>>3
Transgranular (SCC)	4	20	−4	20
Intergranular (SCC #1)	−6.6	18 to 114	1.5 to 3.2	6.4 to 15.6
Intergranular (SCC #2)	−2 to −6	5 to 45	3 to 6	10 to 60

**Table 3:** Parameters obtained by statistical analysis.

Solution	Steels	R_n_ (Ω·cm^2^)	LI	Corrosion Type	Skewness	Corrosion Type	Kurtosis	Corrosion Type
H_2_SO_4_	AerMet 100	5.44 ± 0.1	0.01 ± 0.001	Uniform	0.12 ± 0.03	Uniform	4.71 ± 0.6	Localized
	4340	6.01 ± 0.1	0.002 ± 0.0002	Uniform	0.21 ± 0.04	Uniform	3.05 ± 0.4	Uniform
NaCl	AerMet 100	1599.84 ± 13	0.008 ± 0.0002	Uniform	12.21 ± 0.8	Localized	253.28 ± 2.9	Localized
	4340	87.79 ± 1	0.005 ± 0.0002	Uniform	−0.06 ± 0.003	Uniform	3.07 ± 0.7	Uniform
HCl	AerMet 100	333.22 ± 3	0.0006± 0.00001	Uniform	0.71 ± 0.06	Uniform	6.91 ± 0.4	Localized
	4340	44.55 ± 0.8	0.0005 ± 0.00005	Uniform	0.81 ± 0.08	Uniform	4.26 ± 0.3	Localized

**Table 4:** β intervals to indicate the type of corrosion [31].

Corrosion Type	dB(V)·Decade^−1^		dB(A)·Decade^−1^	
	Minimum	Maximum	Minimum	Maximum
Uniform	0	−7	0	−7
Pitting	−20	−25	−7	−14
Passive	−15	−25	−1	1

**Table 5:** PSD and Z_n_ parameters.

Solution	Steels	*Z_n_* (Ω·cm^2^)	dB(A)·Decade^−1^	Corrosion Type
H_2_SO_4_	AerMet 100	10.20 ± 0.06	−16.29 ± 0.14	Pitting
	4340	11.46 ± 0.04	−12.85 ± 0.08	Pitting
NaCl	AerMet 100	436.74 ± 14	−2.58 ± 0.005	Pitting
	4340	570.15 ± 4.6	−16.04 ± 0.09	Pitting
HCl	AerMet 100	1574.74 ± 15	−10.54 ± 0.56	Pitting
	4340	715.40 ± 5.3	−14.30 ± 0.78	Pitting

## Data Availability

The original contributions presented in this study are included in the article. Further inquiries can be directed to the corresponding authors.
